# Divergent Associations Between Serum Androgens and Ovarian Reserve Markers Revealed in Patients With Polycystic Ovary Syndrome

**DOI:** 10.3389/fendo.2022.881740

**Published:** 2022-06-09

**Authors:** Youran Li, Yanhong Zhai, Lin Li, Yifan Lu, Shaofei Su, Ying Liu, Zhengwen Xu, Mingwei Xin, Qiaoli Zhang, Zheng Cao

**Affiliations:** ^1^ Department of Laboratory Medicine, Beijing Obstetrics and Gynecology Hospital, Capital Medical University, Beijing Maternal and Child Health Care Hospital, Beijing, China; ^2^ Center of Clinical Mass Spectrometry, Beijing Obstetrics and Gynecology Hospital, Capital Medical University, Beijing Maternal and Child Health Care Hospital, Beijing, China; ^3^ Central Laboratory, Beijing Obstetrics and Gynecology Hospital, Capital Medical University, Beijing Maternal and Child Health Care Hospital, Beijing, China; ^4^ Department of Traditional Chinese Medicine, Beijing Obstetrics and Gynecology Hospital, Capital Medical University, Beijing Maternal and Child Health Care Hospital, Beijing, China; ^5^ Department of Human Reproductive Medicine, Beijing Obstetrics and Gynecology Hospital, Capital Medical University, Beijing Maternal and Child Health Care Hospital, Beijing, China

**Keywords:** androgen, ovarian reserve, PCOS, LC-MS/MS, AMH

## Abstract

**Background:**

The role of excess androgen in ovarian reserve remains unclear in patients with polycystic ovary syndrome (PCOS). Our study highlights the associations of serum androgen levels and ovarian reserve markers in PCOS and non-PCOS women.

**Methods:**

Totally 584 menstrual abnormalities women of 20-45 years were retrospectively evaluated at the Beijing Obstetrics and Gynecology Hospital between January 2021 to October 2021. The enrolled patients were classified into two groups: the PCOS group (n=288) and the non-PCOS group (n=296) based on the Rotterdam consensus for PCOS diagnosis. The serum androgens, including testosterone (T), free testosterone (FT, calculated), bioavailable testosterone (Bio-T, calculated), androstenedione (A2), dihydrotestosterone (DHT), dehydroepiandrosterone (DHEA), and dehydroepiandrosterone sulfate (DHEAS) were assessed with an in-house developed liquid chromatography tandem mass spectrometry (LC-MS/MS) method. The associations between the serum androgens and the hormone markers commonly used for evaluating ovarian reserve function, such as anti-mullerian hormone (AMH) and the ratio of luteinizing hormone (LH)/follicle stimulating hormone (FSH) were explored.

**Results:**

The serum T, FT, Bio-T, A2, DHT, DHEA, DHEAS, AMH and LH/FSH of the PCOS group were 51.7 ± 23.2 ng/dL/mL, 8.5 ± 5.0 pg/mL, 210.1 ± 127.7 pg/mL, 1.9 ± 0.8 ng/mL, 0.2 ± 0.1 ng/mL, 6.4 ± 4.2 ng/mL, 2431.0 ± 1030.7 ng/mL, 6.7 ± 3.8 ng/mL, and 1.8 ± 1.4 respectively, which were significantly higher than those in the non-PCOS group (*p*<0.05). In the group of PCOS patients, T and A2 levels were positively associated with AMH in both multivariate linear regression analysis and Pearson’s correlation analysis. Similar but weaker associations were observed in the non-PCOS patients. In the PCOS patients with hyperandrogenemia (HA), the AMH level was significantly higher in the subjects with T increased than in the subjects with non-T androgen(s) increased (A2, DHT, DHEA or DHEAS).

**Conclusions:**

The serum androgen levels are positively associated with ovarian reserve markers in both of the PCOS and the non-PCOS patients in our study. In the PCOS group, the highest AMH level was observed in the subjects with the T elevation subgroup, suggesting that T is more closely related with the increase of AMH when compared with other androgens investigated.

## Introduction

Polycystic ovarian syndrome (PCOS) is the most common endocrine disorder of reproductive-age women, affecting more than 100 million women worldwide (1, 2). The Rotterdam consensus workshop defined PCOS as the presentation of any two of the following conditions: oligo-or anovulation, hyperandrogenemia (HA) and polycystic ovarian morphology (3). PCOS is also frequently associated with obesity, insulin resistance and hyperinsulinemia. Further research revealed that HA with elevated serum concentrations of androgens was the most constant and important diagnostic component of PCOS (4–6), which was believed to play a prominent role in the pathogenesis of this syndrome (7–10).

In women the major circulating androgen precursors and bioactive androgens, in the descending order of serum concentrations are dehydroepiandrosterone sulfate (DHEAS), dehydroepiandrosterone (DHEA), androstenedione (A2), testosterone (T) and dihydrotestosterone (DHT) (11–13). Most testosterone circulates tightly bound to sex hormone-binding globulin (SHBG) or weakly bound to albumin, and the free testosterone is believed to be the metabolically active fraction (14). Increased clinical and laboratory evidence suggest that the measurement of free testosterone is more sensitive and superior to total testosterone for establishing the existence of androgen excess (15–17).

Androgen mediate their actions primarily *via* transcriptionally activating the androgen receptor (AR) (18), which is an important factor of androgen signaling pathway. T and DHT are the only potent bioactive androgens that bind directly to the AR, while DHEAS, DHEA and A2 are pro-androgens requiring conversion to T and/or DHT to exert androgenic effects. Data from clinical studies have demonstrated an important role for androgens in the regulation of ovarian function and female fertility (19). The most common biochemical perturbation in patients with PCOS is the elevation of circulating T and A2 levels (20). Compelling evidence suggested that the major source of androgens in women with PCOS is the ovary (21), although the adrenal gland might also contribute to androgen overproduction in a minority of patients (22, 23).

At present, the clinically and commonly used index of ovarian reserve evaluation are sex hormones in combination with ultrasound based ovarian function assessment. AMH is a member of the transforming growth factor-β superfamily that is produced by growing ovarian antral follicles (5, 24–26). Serum AMH correlates with the total number of antral follicles in the ovaries, and therefore has been proposed as a biomarker for ovarian reserve evaluation (27, 28). In women with PCOS, an alteration in gonadotropin-releasing hormone secretion caused an increase in luteinizing hormone secretion with normal follicle-stimulating hormone secretion, which has been widely accepted as specific endocrine profiles (29). This hormone secretion pattern results in an abnormal LH/FSH ratio, which is considered a valuable marker for evaluating ovarian function and assisting PCOS diagnosis (30). Other commonly used biochemical ovarian reserve markers include but not limited to, estradiol, inhibin B, provocative tests (i.e. clomiphene citrate challenge test). Ultrasonographic measures of ovarian reserve mainly include antral follicle count (AFC) and ovarian volume (31).

Despite the well-established and pivotal roles of androgens in female reproductive function, however, the association between androgens and ovarian reserve function, especially in PCOS remains largely unclear. In this study, we aimed to evaluate the associations between serum androgen profiles measured by liquid chromatography tandem mass spectrometry (LC-MS/MS) and ovarian reserve markers in both PCOS and non-PCOS patients.

## Materials and Methods

From January 2021 to October 2021, the patients (between 20- 45 years) who visited the Department of Gynecological Endocrinology or Human Reproductive Medicine and were tested for hyperandrogenemia by our LC-MS/MS method were initially considered for retrospective analysis. This study was approved by the Ethics Committee of Beijing Obstetrics and Gynecology Hospital, Capital Medical University (approval number: 2022-KY-006-02). Diagnosis of PCOS was established based on the Rotterdam 2003 consensus (3). The PCOS women were either newly detected or treatment naive patients who had never undergone treatment for this syndrome. The non-PCOS women were those who came to our institute for infertility issues and did not present menstrual abnormalities and met the following exclusion criteria: diagnosis of PCOS, previous ovary surgery, ovarian insufficiency, endometriosis, diabetes mellitus, thyroid dysfunction, hyperprolactinemia, Cushing’s syndrome, use of the drugs like spironolactone, aspirin, corticosteroid, and metformin (6).

The serum androgen profiles including testosterone, free testosterone (FT), bioavailable testosterone (Bio-T), androstenedione, dihydrotestosterone, dehydroepiandrosterone, and dehydroepiandrosterone sulfate were quantified with an in-house developed LC-MS/MS method that was established and validated previously (13). Briefly, the serum samples were processed by protein precipitation followed by a solid phase extraction step. The chromatographic separation was achieved with a C18 column, using a linear gradient elution with two mobile phases: 0.02% formic acid in water (phase A) and 0.1% formic acid in methanol (phase B). The target analytes were detected by positive or negative electrospray ionization mode using multiple reaction monitoring (MRM). All participants had their serum levels of anti-mullerian hormone (AMH), luteinizing hormone (LH), follicle stimulating hormone (FSH) and androgen profiles measured on day 2-5 of menstruation period. The serum FSH, LH were determined by the ADVIA Centaur XP Chemiluminescence Analyzer (Simens Healthcare Diagnostics Inc.) and AMH was determined by an enzyme-linked immunoassay (ELISA) method (Guangzhou Kangrun Biological Technology Co., Ltd.). FT was more sensitive than total T in PCOS diagnosis but its direct measurement was cumbersome and laborious. In this study, relatively simple methods were adopted for the estimation of FT and Bio-T, using the parameters of total testosterone, SHBG and albumin (the online calculator website: http://www.issam.ch/freetesto.htm) (32).

Data analysis was performed using statistical software SPSS 23.0. The Kolmogorov-Smirnov test was used to evaluate the normality of the data distribution. Numerical values were expressed as the mean and standard deviation (SD) for variables with normal distribution and as the median and percentiles for nonnormally distributed data. Comparisons between the two groups were performed using the t-test (for normal distribution) or Mann-Whitney-U test (for nonnormal distribution). A one-way ANOVA (“analysis of variance”) was applied to compare the means of three or more independent groups to determine if there is a statistically significant difference between the corresponding population means. Spearman’s correlation was used to evaluate linear relationship between androgen levels and AMH or LH/FSH ratio. Multivariate logistic regression was performed for assessing the strength of the association between androgens and AMH or LH/FSH with age adjustment as a co-variable. All tests were two-sided, and *p*<0.05 was considered statistically significant.

## Results

Of the 980 subjects screened, 288 PCOS and 296 non-PCOS women meeting the corresponding inclusion and exclusion criteria were included for subsequent analysis. The subjects’ age, serum androgen profiles, AMH, and LH/FSH ratios were listed and compared in **Table 1**. As shown in **Supplementary Figure 1**, the levels of serum androgens including T, FT, Bio-T, A2, DHT, DHEA, and DHEAS, were found significantly elevated in the PCOS group than those in the non-PCOS group. For the ovarian reserve function markers, both AMH and LH/FSH ratio in the PCOS patients were 2-3 times higher than in the non-PCOS controls, which was coincidently in line with a younger average age observed in the PCOS group (**Table 1** and **Supplementary Figure 1**).

**Table 1:** The age, androgens, AMH and LH/FSH ratio of women in the PCOS and the non-PCOS group.

Characteristics	PCOS (n=288)	Non-PCOS (n=296)	*p* value
Age (years)	30.2 ± 4.8	33.3 ± 6.4	<0.001
T (ng/dL)	51.7 ± 23.2	25.1 ± 10.0	<0.001
FT (pg/mL)	8.5 ± 5.0	3.7 ± 1.7	<0.001
Bio-T (pg/mL)	210.1 ± 127.7	89.2 ± 43.1	<0.001
A2 (ng/mL)	1.9 ± 0.8	1.0 ± 0.4	<0.001
DHT (ng/mL)	0.2 ± 0.1	0.2 ± 0.2	0.032
DHEA (ng/mL)	6.4 ± 4.2	4.5 ± 2.4	<0.001
DHEAS (ng/mL)	2431.0 ± 1030.7	1733.3 ± 716.4	<0.001
AMH (ng/mL)	6.7 ± 3.8	1.9 ± 2.4	<0.001
LH/FSH	1.8 ± 1.4	0.9 ± 0.7	<0.001

Values are presented as mean ± standard deviation. T, testosterone; FT, free testosterone; Bio-T, bioavailable testosterone; A2, androstenedione; DHT, dihydrotestosterone; DHEA, dehydroepiandrosterone; DHEAS, dehydroepiandrosterone sulfate; AMH, anti-mullerian hormone; LH, and the ratio of luteinizing hormone; FSH, follicle stimulating hormone.

In a multivariate linear regression model adjusted for age which was a well-known confounding factor for ovarian function assessment, both serum T and A2 levels were positively associated with serum AMH in the PCOS group (**Table 2**). Specifically, one unit increase in T or A2 levels (ng/mL) were associated with an increase of 0.039 or 1.122 units of AMH (ng/mL), respectively. Similar positive association was observed in the non-PCOS group between androgens (FT, BIO-T, and A2) and AMH levels. However, variable or even contradictory associations were seen between androgens and the LH/FSH ratio within the non-PCOS patients: testosterone and its derivatives (FT, Bio-T) were positively related with LH/FSH, but DHEA and DHEAS were negatively associated.

**Table 2:** Multivariate linear regression studies between androgens and AMH or LH/FSH.

Androgens	PCOS group				Non-PCOS group			
	AMH		LH/FSH		AMH		LH/FSH	
	β	*p* value	β	*p* value	β	*p* value	β	*p* value
T	0.039	0.001	0.008	0.095	0.022	0.117	0.012	0.006
FT	0.091	0.094	0.021	0.313	0.204	0.023	0.062	0.025
Bio-T	0.004	0.081	0.001	0.209	0.007	0.038	0.003	0.02
A2	1.122	0.001	0.49	<0.001	1.372	<0.001	0.264	0.013
DHT	0.85	0.704	-0.124	0.883	-0.452	0.573	-0.166	0.491
DHEAS	-0.0003	0.213	0.0001	0.46	0.0001	0.554	-0.0002	0.009
DHEA	-0.047	0.405	-0.021	0.362	-0.105	0.066	-0.049	0.006

To further examine the relationship between serum androgens with indicators of ovarian function, Pearson correlation analysis was performed. As shown in **Supplementary Figure 2** and **Table 3**, positive correlations between T, FT, Bio-T, A2 and AMH or LH/FSH ratio were observed in both of the PCOS and the non-PCOS groups (*p<*0.05). In the non-PCOS group, interestingly, the serum DHEAS level was positively correlated with AMH (r=0.164, *p*=0.006) but negative correlated with LH/FSH ratio (r=-0.136, *p*=0.031) (**Table 3**).

**Table 3:** Pearson correlation analysis between androgens and AMH or LH/FSH.

Androgens	PCOS group				Non-PCOS group			
	AMH		LH/FSH		AMH		LH/FSH	
	r	*p* value	r	*p* value	r	*p* value	r	*p* value
T	0.301	<0.001	0.198	0.003	0.12	0.049	0.267	<0.001
FT	0.187	0.009	0.235	<0.001	0.13	0.032	0.249	<0.001
Bio-T	0.185	0.010	0.255	<0.001	0.13	0.032	0.239	<0.001
A2	0.319	<0.001	0.396	<0.001	0.267	<0.001	0.314	<0.001
DHT	0.049	0.496	0.06	0.375	-0.027	0.656	-0.048	0.453
DHEAS	-0.005	0.948	0.031	0.645	0.164	0.006	-0.136	0.031
DHEA	-0.04	0.582	-0.061	0.367	-0.059	0.332	-0.119	0.06

Next, the serum levels of AMH and the LH/FSH ratio were compared between the three subgroups of the PCOS subjects (**Table 4**): T subgroup (HA with T elevated), non-T subgroup (HA with other androgens elevated but T, such as A2, DHT, DHEA or DHEAS), non-HA subgroup (no androgen elevated). As shown in **Table 4**, the AMH was higher in the T subgroup than that in the non-T and non-HA subgroups (*p<*0.05); but there was no significant difference between non-T and non-HA subgroups for AMH level. There was no statistical difference for the LH/FSH ratio among the three groups compared in **Table 4** by one-way ANOVA analysis.

**Table 4:** Comparison of the serum AMH and LH/FSH ratio between the three subgroups of the PCOS patients.

	ANOVA		Multiple comparisons		
	F value	P value	Subgroups	Mean ± Sd.	*p* value
AMH (ng/mL)	3.38	0.04	T	7.23 ± 3.61	0.039 (*vs* non-T)
			Non-T	5.86 ± 3.98	0.746 (*vs* non-HA)
			Non-HA	5.73 ± 3.82	0.045 (*vs* T)
LH/FSH	0.47	0.63	T	1.82 ± 1.28	0.962 (*vs* non-T)
			Non-T	1.83 ± 1.39	0.396 (*vs* non-HA)
			Non-HA	1.57 ± 1.86	0.356 (*vs* T)

T subgroup (hyperandrogenemia (HA) with T elevated), non-T subgroup (HA with other androgens elevated but T, such as A2, DHT, DHEA or DHEAS), non-HA subgroup (no androgen elevated). A one-way analysis of variance (ANOVA), followed by a LSD (Least Significant Difference) test, was used in comparison among three groups.

## Discussion

In this study, as expected, serum androgen levels were significantly increased in the women with PCOS compared to the non-PCOS controls (**Table 1** and **Supplementary Figure 1**). According to the report by Jonard and Dewailly, hyperandrogenism had become the core event of PCOS and had an essential impact in disturbing folliculogenesis (33, 34). As shown in **Supplementary Figure 2**, both in the PCOS and non-PCOS groups, there were positive correlations between T, FT, Bio-T, A2 and AMH or LH/FSH, suggesting that those androgens play a vital part in maintaining the health of the female reproductive system. Serum levels of androgens, AMH and FSH presented complex interactions during folliculogenesis (35). Specifically, with its receptors enhanced by androgens, FSH would indirectly stimulate the AMH production through granulosa cell (GC) proliferation. However, a stimulating effect from androgens on AMH production remains controversial as indicated by both *in vivo* and *in vitro* experiments. Further, accumulated evidence showed that elevated androgens may lead to abnormal frequency and amplitude of gonadotropin-releasing hormone (GnRH) release, resulting in the production of LH from the pituitary gland and therefore increased LH/FSH ratio (36). As the biology holds true regardless a patient’s diagnosis, positive associations were observed in our study between androgens and ovarian reserve parameters in both PCOS and non-PCOS women. Recently, a large retrospective cross-sectional study by Lin et al. in 1935 infertile women of which only 9.6% were diagnosed with PCOS, showed a strong positive association between serum testosterone and AMH levels (37).

Similar observations were made in the multivariate linear regression model adjusted for age in **Table 2**, where both T and A2 were positively associated with AMH or LH/FSH ratio. However, a negative correlation between DHEA/DHEAS and LH/FSH ratio, was noted in the non-PCOS patients whose chief complaint was infertility (**Table 3**). This may be explained by the fact that DHEA/DHEAS affects early follicle maturation and LH/FSH ratio by increasing expression of androgen receptor (AR) and FSH receptor (FSHR) in granulosa cells of in diminished ovarian reserve women (38).

More importantly, the increase of AMH was majorly contributed by testosterone elevation, not other androgens measured simultaneously, as suggested by **Table 4**. The causes of hyperandrogenism in PCOS patients are multifactorial and involve both of the hypothalamus-pituitary-ovarian axis and adrenal axis. PCOS patients with increased secretion of GnRH and LH can enhance follicles activity of androgen synthesis rate limiting enzyme P450c17a in membrane cells. In addition, the insulin resistance is enhanced in PCOS patients, which can directly stimulate the production of the ovarian androgen. It can also increase free testosterone levels by inhibiting the synthesis of SHBG in liver (8).

PCOS is characterized by an increased number of ovarian follicles at all growing stages. This increase is particularly seen in the pre-antral and small antral follicles. It is precisely those follicles that mainly produce AMH. Consistent with our research, several studies have shown that serum AMH was positively correlated with androgen levels and the level of AMH was found 2-4 folds higher in women with PCOS than in healthy women (39). This elevated serum AMH level was considered a reflection of the increased stock of pre-antral and small antral follicles within PCOS (40). In addition, it could also result from an increased production of AMH per follicle, suggesting a probable over-expression of AMH by the GCs from antral follicles in PCOS women. On the other hand, an indirect effect of androgens leads to an increase in the number of FSH receptors (FSHR) and/or estradiol receptors alpha (41). Consequently, the stimulating effect of FSH on AMH expression that occurs in small growing follicles from normal ovaries would be amplified in PCOS. The excessive level of AMH reduces the sensitivity of FSH stimulated follicles by reducing the expression of aromatase, inhibits the maturation of follicles, interferes with the growth and differentiation of antral follicles, and prevents the conversion of androgens to estrogen, aggravating hyperandrogenism. However, it remains controversial whether androgens can directly stimulate AMH secretion. For example, an androgen-inhibitory effect of androgens on the secretion of AMH by Sertoli cells in men has been clearly demonstrated (42). The contradictory results may be explained by the great variability of the models used (i.e., different animal species, cell types, analysis methods).

The interaction between AMH and androgens are complicated and not sufficiently addressed. In a cross-sectional study by Lv et al. (43), it was reported that T was positive correlated with AMH level in both diminished ovarian reserve (DOR) and PCOS which are manifested with insufficient AMH and excessive AMH respectively, suggesting total testosterone plays an important role in follicular growth. Zhang et al. (44) showed that testosterone, not DHT, caused an increase in AMH mRNA in granulosa cells from mouse antral follicles, implying divert roles of different androgens in AMH overexpression. In another interesting clinical study in DOR women, there was no statistically improvement in the ovarian reserve markers (AFC, AMH or FSH) after DHEA treatment; however, DHEA supplementation did upregulate the expression of AR and FSHR in GC cells, suggesting an alternative mechanism that non-T androgens such as DHEA may contribute to PCOS pathogenesis (38). Therefore, in PCOS patients, testosterone seems to be more closely related to ovarian reserve markers evaluated by serum AMH level, which may provide alternative insight for the future mechanism studies of PCOS.

### Strengths and Limitations

The serum androgens were measured by a previously validated LC-MS/MS, which is the recommended and considered as “gold-standard” methodology for androgen quantitation in female (7, 14). The application of LC-MS/MS warrants better accuracy of androgen measurements compared with conventional immunoassays. Significantly, in the PCOS patients, the highest AMH level was observed in the subjects with the T elevation subgroup, suggesting that T is more closely related with the increase of AMH when compared with other androgens investigated.

A few limitations exist in this study. First, as a multifactorial and complex disease, in addition to age of subjects, many other factors could have impact on the relationship between androgens and ovarian reserve markers. However, these contributing factors, such as body weight, BMI, insulin resistance and oral glucose tolerance test (OGTT) status, and AFC, unfortunately are not retrievable due to the retrospective nature of the study. Second, lack of pregnancy or *in vitro* fertilization (IVF) outcome limits its application in clinical practice for the interaction between androgens and ovarian reserve markers.

## Conclusion

In present study, the serum androgen levels were found significantly increased in women with PCOS compared to the non-PCOS controls. Both in the PCOS and the non-PCOS groups, there were positive correlations between T, FT, Bio-T, A2 and AMH or LH/FSH. Last but not least, the AMH level in the T-elevation subgroup of PCOS patients was significantly higher than that in the non-T elevation or the non-HA subgroup. This interesting finding may be used to predict the ovarian response in patients receiving controlled ovarian hyperstimulation. However, whether testosterone plays a different role in the pathogenesis of PCOS requires further clinical and mechanistic studies.

## Data Availability Statement

The raw data supporting the conclusions of this article will be made available by the authors, without undue reservation.

## Ethics Statement

This study was approved by the Ethics Committee of Beijing Obstetrics and Gynecology Hospital, Capital Medical University (approval number: 2022-KY-006-02). Written informed consent for participation was not required for this study in accordance with the national legislation and the institutional requirements.

## Author Contributions

All authors have certified the author list and the contribution description. All authors have read and approved the submitted manuscript and any substantially modified version of the manuscript. Contribution to work: ZC, QZ, and MX were involved in study conception and design and patient recruitment; YRL, YL, and ZX. were involved in performing the experiments, and data acquisition, analysis and interpretation; YZ, LL, QZ, MX, and ZC drafted the article and critically reviewed and approved the final article; YFL and SS contributed to the statistical analysis and figure preparation.

## Funding

This study was supported by the Specialized Youth Foundation Project of Beijing Obstetrics and Gynecology Hospital, Capital Medical University (No. FCYYQN-202102). The funding body did not take part in the design of the study, the collection, analysis and interpretation of the data, or manuscript writing.

## Conflict of Interest

The authors declare that the research was conducted in the absence of any commercial or financial relationships that could be construed as a potential conflict of interest.

## Publisher’s Note

All claims expressed in this article are solely those of the authors and do not necessarily represent those of their affiliated organizations, or those of the publisher, the editors and the reviewers. Any product that may be evaluated in this article, or claim that may be made by its manufacturer, is not guaranteed or endorsed by the publisher.

## Supplementary Material

Supplementary Figure 1 Box plots representing the serum levels of testosterone (T), free testosterone (FT), bioavailable testosterone (Bio-T), androstenedione (A2), dihydrotestosterone (DHT), dehydroepiandrosterone (DHEA), dehydroepiandrosterone sulfate (DHEAS), anti-mullerian hormone (AMH) and the ratio of luteinizing hormone (LH)/follicle stimulating hormone (FSH) in the PCOS and non-PCOS groups. Asterisk (*) indicates p<0.05.

Supplementary Figure 2 Pearson’s correlations between serum AMH level or LH/FSH ratio and androgens (T, FT, Bio-T, A2, DHT and DHEAS) in both of the PCOS and non-PCOS patients.

## References

[1] Azziz R Carmina E Chen Z Dunaif A Laven JS Legro RS. Polycystic Ovary Syndrome. Nat Rev Dis Primers (2016) 2:16057. doi: 10.1038/nrdp.2016.57 27510637

[2] Tay CT Joham AE Hiam DS Gadalla MA Pundir J Thangaratinam S. Pharmacological and Surgical Treatment of Nonreproductive Outcomes in Polycystic Ovary Syndrome: An Overview of Systematic Reviews. Clin Endocrinol (Oxf) (2018) 89(5):535–53. doi: 10.1111/cen.13753 29846959

[3] Rotterdam ESHRE/ASRM-Sponsored PCOS Consensus Workshop Group. Revised 2003 Consensus on Diagnostic Criteria and Long-Term Health Risks Related to Polycystic Ovary Syndrome (PCOS). Hum Reprod (2004) 19(1):41–7. doi: 10.1093/humrep/deh098 14688154

[4] Escobar-Morreale HF. Polycystic Ovary Syndrome: Definition, Aetiology, Diagnosis and Treatment. Nat Rev Endocrinol (2018) 14(5):270–84. doi: 10.1038/nrendo.2018.24 29569621

[5] Tal R Seifer DB. Ovarian Reserve Testing: A User’s Guide. Am J Obstet Gynecol (2017) 217(2):129–40. doi: 10.1016/j.ajog.2017.02.027 28235465

[6] Nikbakht R Zargar M Moramezi F Ziafat M Tabesh H Sattari AR. Insulin Resistance and Free Androgen as Predictors for Ovarian Hyperstimulation Syndrome in Non-PCOS Women. Horm Metab Res (2020) 52(2):104–8. doi: 10.1055/a-1079-5342 31975364

[7] Azziz R Carmina E Dewailly D Diamanti-Kandarakis E Escobar-Morreale HF Futterweit W. Positions Statement: Criteria for Defining Polycystic Ovary Syndrome as a Predominantly Hyperandrogenic Syndrome: An Androgen Excess Society Guideline. J Clin Endocrinol Metab (2006) 91(11):4237–45. doi: 10.1210/jc.2006-0178 16940456

[8] Rosenfield RL Ehrmann DA. The Pathogenesis of Polycystic Ovary Syndrome (PCOS): The Hypothesis of PCOS as Functional Ovarian Hyperandrogenism Revisited. Endocr Rev (2016) 37(5):467–520. doi: 10.1210/er.2015-1104 27459230 PMC5045492

[9] Goodman NF Cobin RH Futterweit W Glueck JS Legro RS Carmina E. American Association of Clinical Endocrinologists, American College of Endocrinology, and Androgen Excess and PCOS Society Disease State Clinical Review: Guide to the Best Practices In the Evaluation and Treatment of Polycystic Ovary Syndrome - Part 2. Endocr Pract (2015) 21(12):1415–26. doi: 10.4158/EP15748.DSCPT2 26642102

[10] Carmina E. Ovarian and Adrenal Hyperandrogenism. Ann N Y Acad Sci (2006) 1092:130–7. doi: 10.1196/annals.1365.011 17308139

[11] Yildiz BO Bozdag G Yapici Z Esinler I Yarali H. Prevalence, Phenotype and Cardiometabolic Risk of Polycystic Ovary Syndrome Under Different Diagnostic Criteria. Hum Reprod (2012) 27(10):3067–73. doi: 10.1093/humrep/des232 22777527

[12] Azziz R Carmina E Dewailly D Diamanti-Kandarakis E Escobar-Morreale HF Futterweit W. The Androgen Excess and PCOS Society Criteria for the Polycystic Ovary Syndrome: The Complete Task Force Report. Fertil Steril (2009) 91(2):456–88. doi: 10.1016/j.fertnstert.2008.06.035 18950759

[13] Cao Z Lu Y Cong Y Liu Y Li Y Wang H. Simultaneous Quantitation of Four Androgens and 17-Hydroxyprogesterone in Polycystic Ovarian Syndrome Patients by LC-MS/MS. J Clin Lab Anal (2020) 34(12):e23539. doi: 10.1002/jcla.23539 32820576 PMC7755789

[14] Goodman NF Cobin RH Futterweit W Glueck JS Legro RS Carmina E. American Association of Clinical Endocrinologists, American College of Endocrinology, and Androgen Excess and PCOS Society Disease State Clinical Review: Guide to the Best Practices In the Evaluation and Treatment of Polycystic Ovary Syndrome–Part 1. Endocr Pract (2015) 21(11):1291–300. doi: 10.4158/EP15748.DSC 26509855

[15] Shea JL Wong PY Chen Y. Free Testosterone: Clinical Utility and Important Analytical Aspects of Measurement. Adv Clin Chem (2014) 63:59–84. doi: 10.1016/b978-0-12-800094-6.00002-9 24783351

[16] Khattak M Usman R Sultana N Khattak A. Comparison Of Free Androgen Index In Polycystic Ovary Syndrome And Non-Polycystic Ovary Syndrome Infertile Patients. J Ayub Med Coll Abbottabad (2021) 33(4):577–81. 35124911

[17] Karakas SE. New Biomarkers for Diagnosis and Management of Polycystic Ovary Syndrome. Clin Chim Acta (2017) 471:248–53. doi: 10.1016/j.cca.2017.06.009 28624501

[18] van Houten EL Kramer P McLuskey A Karels B Themmen AP Visser JA. Reproductive and Metabolic Phenotype of a Mouse Model of PCOS. Endocrinology (2012) 153(6):2861–9. doi: 10.1210/en.2011-1754 22334715

[19] Dewailly D Robin G Peigne M Decanter C Pigny P Catteau-Jonard S. Interactions Between Androgens, FSH, Anti-Müllerian Hormone and Estradiol During Folliculogenesis in the Human Normal and Polycystic Ovary. Hum Reprod Update (2016) 22(6):709–24. doi: 10.1093/humupd/dmw027 27566840

[20] Zhang TT Yang XL Yang SX Shang J Xue Q Zhang X. [Analysis of Clinical Features and Etiological Diagnostic Indices of Reproductive Age Women With Hyperandrogenism]. Zhonghua Yi Xue Za Zhi (2022) 102(6):412–7. doi: 10.3760/cma.j.cn112137-20210728-01683 35144340

[21] Li A Zhang L Jiang J Yang N Liu Y Cai L. Follicular Hyperandrogenism and Insulin Resistance in Polycystic Ovary Syndrome Patients With Normal Circulating Testosterone Levels. J BioMed Res (2017) 32(3):208–14. doi: 10.7555/JBR.32.20170136 PMC6265400 29760297

[22] Moran C Reyna R Boots LS Azziz R. Adrenocortical Hyperresponsiveness to Corticotropin in Polycystic Ovary Syndrome Patients With Adrenal Androgen Excess. Fertil Steril (2004) 81(1):126–31. doi: 10.1016/j.fertnstert.2003.07.008 14711555

[23] Kumar A Woods KS Bartolucci AA Azziz R. Prevalence of Adrenal Androgen Excess in Patients With the Polycystic Ovary Syndrome (PCOS). Clin Endocrinol (Oxf) (2005) 62(6):644–9. doi: 10.1111/j.1365-2265.2005.02256.x 15943823

[24] Barbarino-Monnier P. Insuffisance Ovarienne Prématurée [Premature Ovarian Failure]. J Gynecol Obstet Biol Reprod (Paris) (2000) 29(3):316–8. 10804382

[25] Moolhuijsen LME Visser JA. Anti-Müllerian Hormone and Ovarian Reserve: Update on Assessing Ovarian Function. J Clin Endocrinol Metab (2020) 105(11):3361–73. doi: 10.1210/clinem/dgaa513 PMC7486884 32770239

[26] Lew R. Natural History of Ovarian Function Including Assessment of Ovarian Reserve and Premature Ovarian Failure. Best Pract Res Clin Obstet Gynaecol (2019) 55:2–13. doi: 10.1016/j.bpobgyn.2018.05.005 30420162

[27] Dewailly D Andersen CY Balen A Broekmans F Dilaver N Fanchin R. The Physiology and Clinical Utility of Anti-Mullerian Hormone in Women. Hum Reprod Update (2014) 20(3):370–85. doi: 10.1093/humupd/dmt062 24430863

[28] Depmann M van Disseldorp J Broer SL Eijkemans MJ Laven JS Visser JA. Fluctuations in Anti-Müllerian Hormone Levels Throughout the Menstrual Cycle Parallel Fluctuations in the Antral Follicle Count: A Cohort Study. Acta Obstet Gynecol Scand (2016) 95(7):820–8. doi: 10.1111/aogs.12886 26919173

[29] Blank SK McCartney CR Marshall JC. The Origins and Sequelae of Abnormal Neuroendocrine Function in Polycystic Ovary Syndrome. Hum Reprod Update (2006) 12(4):351–61. doi: 10.1093/humupd/dml017 16670102

[30] Le MT Le VNS Le DD Nguyen VQH Chen C Cao NT. Exploration of the Role of Anti-Mullerian Hormone and LH/FSH Ratio in Diagnosis of Polycystic Ovary Syndrome. Clin Endocrinol (Oxf) (2019) 90(4):579–85. doi: 10.1111/cen.13934 30636332

[31] Practice Committee of the American Society for Reproductive Medicine Electronic address: asrm@asrm.org Practice Committee of the American Society for Reproductive Medicine. Testing and Interpreting Measures of Ovarian Reserve: A Committee Opinion. Fertil Steril (2020) 114(6):1151–7. doi: 10.1016/j.fertnstert.2020.09.134 33280722

[32] Vermeulen A Verdonck L Kaufman JM. A Critical Evaluation of Simple Methods for the Estimation of Free Testosterone in Serum. J Clin Endocrinol Metab (1999) 84(10):3666–72. doi: 10.1210/jcem.84.10.6079 10523012

[33] Catteau-Jonard S Dewailly D. Pathophysiology of Polycystic Ovary Syndrome: The Role of Hyperandrogenism. Front Horm Res (2013) 40:22–7. doi: 10.1159/000341679 24002402

[34] Franks S Berga SL. Does PCOS Have Developmental Origins? Fertil Steril (2012) 97(1):2–6. doi: 10.1016/j.fertnstert.2011.11.029 22192134 PMC3263824

[35] Dewailly D Barbotin AL Dumont A Catteau-Jonard S Robin G. Role of Anti-Müllerian Hormone in the Pathogenesis of Polycystic Ovary Syndrome. Front Endocrinol (Lausanne) (2020) 11:641. doi: 10.3389/fendo.2020.00641 33013710 PMC7509053

[36] De Leo V la Marca A Petraglia F. Insulin-Lowering Agents in the Management of Polycystic Ovary Syndrome. Endocr Rev (2003) 24(5):633–67. doi: 10.1210/er.2002-0015 14570747

[37] Lin LT Li CJ Tsui KH. Serum Testosterone Levels are Positively Associated With Serum Anti-Mullerian Hormone Levels in Infertile Women. Sci Rep (2021) 11(1):6336. doi: 10.1038/s41598-021-85915-x 33737663 PMC7973568

[38] Hu Q Hong L Nie M Wang Q Fang Y Dai Y. The Effect of Dehydroepiandrosterone Supplementation on Ovarian Response is Associated With Androgen Receptor in Diminished Ovarian Reserve Women. J Ovarian Res (2017) 10(1):32. doi: 10.1186/s13048-017-0326-3 28472976 PMC5418866

[39] Dewailly D Pigny P Soudan B Catteau-Jonard S Decanter C Poncelet E. Reconciling the Definitions of Polycystic Ovary Syndrome: The Ovarian Follicle Number and Serum Anti-Müllerian Hormone Concentrations Aggregate With the Markers of Hyperandrogenism. J Clin Endocrinol Metab (2010) 95(9):4399–405. doi: 10.1210/jc.2010-0334 20610596

[40] Pierre A Peigné M Grynberg M Arouche N Taieb J Hesters L. Loss of LH-Induced Down-Regulation of Anti-Müllerian Hormone Receptor Expression may Contribute to Anovulation in Women With Polycystic Ovary Syndrome. Hum Reprod (2013) 28(3):762–9. doi: 10.1093/humrep/des460 23321213

[41] Dumont A Robin G Dewailly D. Anti-Müllerian Hormone in the Pathophysiology and Diagnosis of Polycystic Ovarian Syndrome. Curr Opin Endocrinol Diabetes Obes (2018) 25(6):377–84. doi: 10.1097/MED.0000000000000445 30299432

[42] Rey R Lukas-Croisier C Lasala C Bedecarrás P. AMH/MIS: What We Know Already About the Gene, the Protein and its Regulation. Mol Cell Endocrinol (2003) 211(1-2):21–31. doi: 10.1016/j.mce.2003.09.007 14656472

[43] Lv PP Jin M Rao JP Chen J Wang LQ Huang CC. Role of Anti-Müllerian Hormone and Testosterone in Follicular Growth: A Cross-Sectional Study. BMC Endocr Disord (2020) 20(1):101. doi: 10.1186/s12902-020-00569-6 32641160 PMC7341602

[44] Zhang Y Wang SF Zheng JD Zhao CB Zhang YN Liu LL. Effects of Testosterone on the Expression Levels of AMH, VEGF and HIF-1伪 in Mouse Granulosa Cells. Exp Ther Med (2016) 12(2):883–8. doi: 10.3892/etm.2016.3436 PMC4950823 27446291

