# Supplementary figures and images for: Divergent Associations Between Serum Androgens and Ovarian Reserve Markers Revealed in Patients With Polycystic Ovary Syndrome
Source: Front Endocrinol (Lausanne). 2022 Jun 9;13:881740. doi: 10.3389/fendo.2022.881740 (PMC9218193; doi:10.3389/fendo.2022.881740)

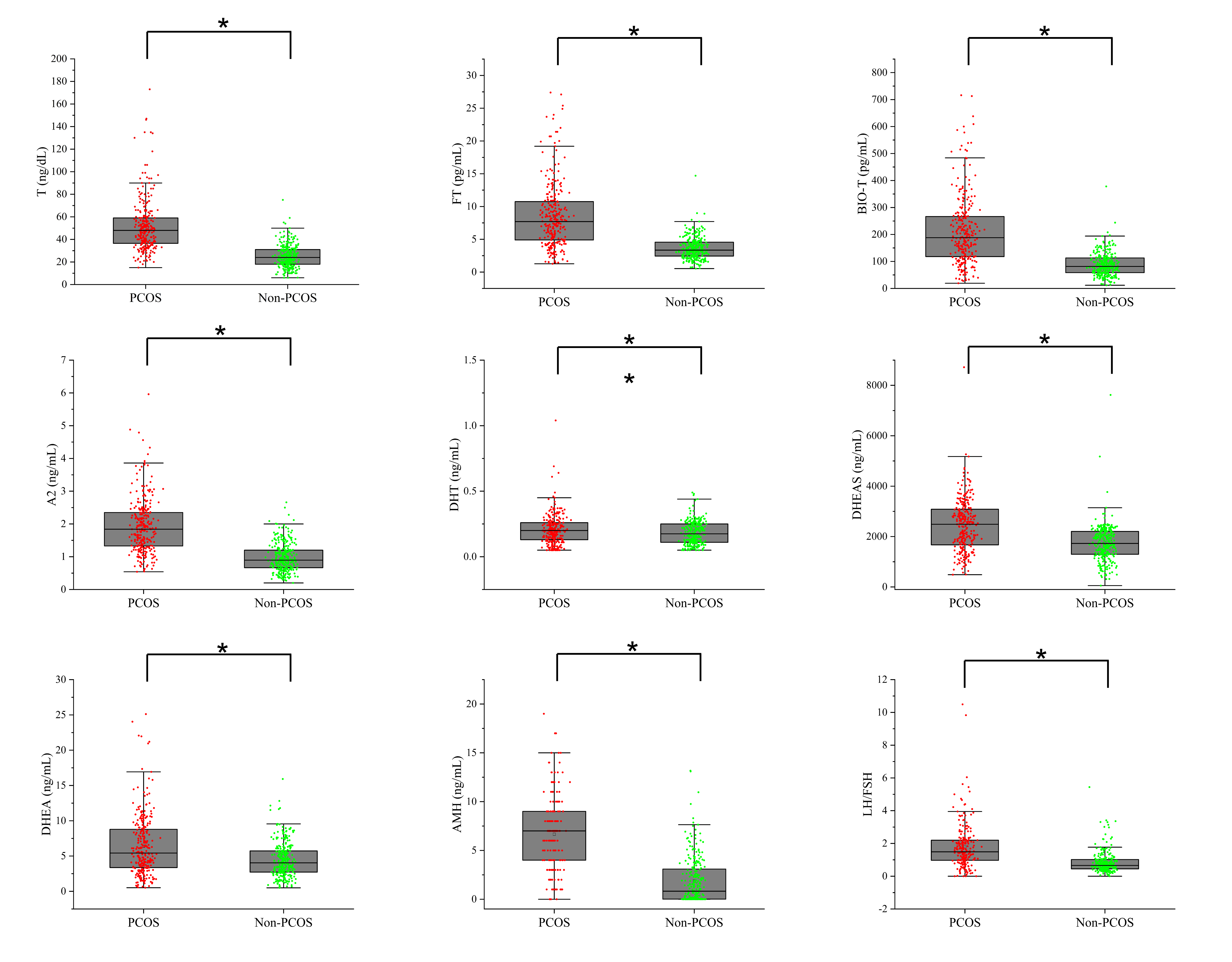

Supplement: Supplementary Figure 1 — Box plots representing the serum levels of testosterone (T), free testosterone (FT), bioavailable testosterone (Bio-T), androstenedione (A2), dihydrotestosterone (DHT), dehydroepiandrosterone (DHEA), dehydroepiandrosterone sulfate (DHEAS), anti-mullerian hormone (AMH) and the ratio of luteinizing hormone (LH)/follicle stimulating hormone (FSH) in the PCOS and non-PCOS groups. Asterisk (*) indicates p<0.05. [file Image_1.tif]

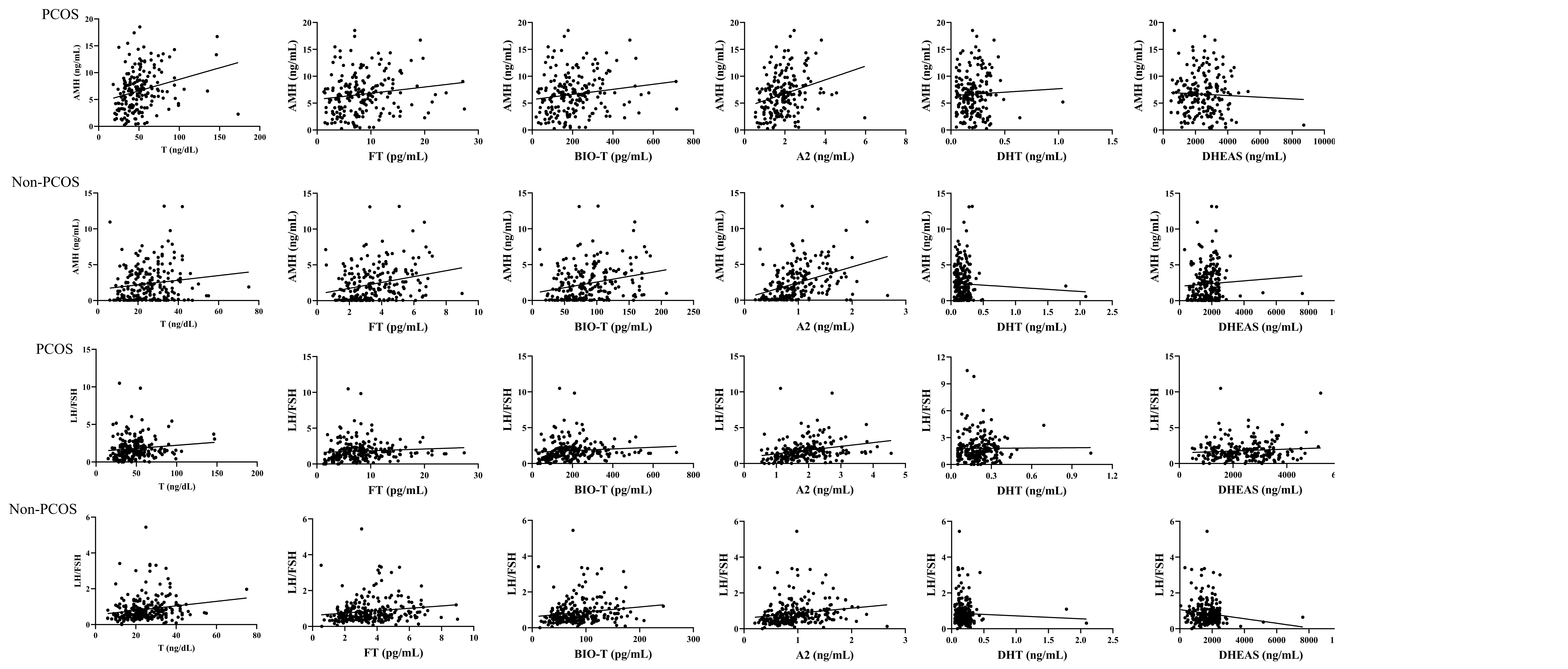

Supplement: Supplementary Figure 2 — Pearson’s correlations between serum AMH level or LH/FSH ratio and androgens (T, FT, Bio-T, A2, DHT and DHEAS) in both of the PCOS and non-PCOS patients. [file Image_2.tif]
